# Radiopharmaceuticals for Pancreatic Cancer: A Review of Current Approaches and Future Directions

**DOI:** 10.3390/ph17101314

**Published:** 2024-10-01

**Authors:** Sara Calistri, Giuseppe Ottaviano, Alberto Ubaldini

**Affiliations:** 1 Department of Pharmacy and Biotechnology, Alma Mater Studiorum, University of Bologna, 40126 Bologna, Italy; 2 ENEA, Italian National Agency for New Technologies, Energy and Sustainable Economic Development, C.R. Bologna, Via Martiri di Monte Sole 4, 40129 Bologna, Italy; giuseppe.ottaviano@enea.it (G.O.); alberto.ubaldini@enea.it (A.U.)

**Keywords:** radiopharmaceuticals, pancreatic cancer, radionuclides, PRRT, NETs

## Abstract

The poor prognosis of pancreatic cancer requires novel treatment options. This review examines the evolution of radiopharmaceuticals in the treatment of pancreatic cancer. Established strategies such as peptide receptor radionuclide therapy (PRRT) offer targeted and effective treatment, compared to conventional treatments. However, there are currently no radiopharmaceuticals approved for the treatment of pancreatic cancer in Europe, which requires further research and novel approaches. New radiopharmaceuticals including radiolabeled antibodies, peptides, and nanotechnological approaches are promising in addressing the challenges of pancreatic cancer therapy. These new agents may offer more specific targeting and potentially improve efficacy compared to traditional therapies. Further research is needed to optimize efficacy, address limitations, and explore the overall potential of these new strategies in the treatment of this aggressive and harmful pathology.

## 1. Introduction

Pancreatic cancer is a highly malignant tumor of the digestive tract, which is difficult to treat and seriously endangers human life and health [1]. It accounts for almost as many deaths (466,000) as cases (496,000) because of its poor prognosis, and is the seventh leading cause of cancer death in both sexes [2]. According to Ferlay et al. in 2016, pancreatic cancer is projected to become the third leading cause of cancer death in the EU by 2025, surpassing colorectal cancer and accounting for 25% of all cancer deaths [3]. The World Health Organization (WHO) in 2020 revealed that among people of all ages and considering both sexes, pancreatic cancer has been identified as the third leading cancer-related cause of death in different countries including Italy, Germany, Austria, Czechia, Hungary, Malta, Spain, Switzerland and USA [4]. For some other countries, in particular, Finland, Qatar, Benin and Guadeloupe, pancreatic cancer was revealed to be the second leading cause of death among all the different types of cancer in 2020 (among females aged ≥70 years [4].

Tumors of pancreas are divided into two groups: (1) Non-endocrine pancreas tumors (2) Endocrine pancreas tumors [5,6]. Non-endocrine pancreas tumors are categorized as benign and malignant [5,6]. Benign non-endocrine tumors of the pancreas: adenoma, cystadenoma, lipoma, fibroma, hemangioma, lymphangioma and neuroma [5,6]. Malignant tumors of the pancreas have different histological features, i.e., (1) ductal adenocarcinoma (2) cystadenocarcinoma and (3) other (sarcomas, metastatic, etc.) malignant tumors [5,6].

Pancreatic neuroendocrine neoplasms originate from pancreatic endocrine cells, differently from pancreatic adenocarcinoma which originates from the epithelial cells of the pancreatic ducts [7]. It is worth underlining the histological difference between these two types of neoplasms since they have different progression and, consequently, different treatments. The first type (NETs), even if in some cases an aggressive course of the disease can occur, usually translates into a better prognosis and a higher probability of survival compared to the second type reported (PDAC), which has a much more aggressive course, with rapidly infiltrative growth [7,8,9].

The estimates underscore the pressing necessity for novel and groundbreaking treatment approaches to benefit patients with this deadly disease. Numerous initiatives are currently underway to modify the disease’s progression, to keep pace with the improved outcomes that have been observed in other malignancies [10].

The conventional therapies for pancreatic cancer (PC) typically involve surgery, chemotherapy, radiotherapy, and palliative care. However, there has been an increasing focus on targeted therapy, immunotherapy, and microbial therapy in recent years, which may be integrated with traditional approaches to treat PC in the future [11]. The stage of the disease is a crucial determinant of its treatment and prognosis [12,13,14,15], indeed the treatment options are selected depending on the stage of PC in a multidisciplinary approach [16]. For patients with local or regional disease, the treatment and prognosis depend on the resectability of the tumor; incomplete resection can have a detrimental effect on survival, similar to those presenting with metastatic disease [12,13,14,15]. With curative surgery, the recurrence rates of pancreatic cancer remain notably high. Consequently, chemotherapy becomes the inevitable choice post-surgery for patients. However, the overall prognosis for patients undergoing adjuvant chemotherapy remains grim due to the low vasculature and the development of an immunosuppressive cancer-associated microenvironment surrounding the pancreas [17]. In a patient with unresectable or locally advanced pancreatic cancer (LAPC), radiotherapy with or without chemotherapy remains an important modality in achieving local control [18].

**Figure 1:**
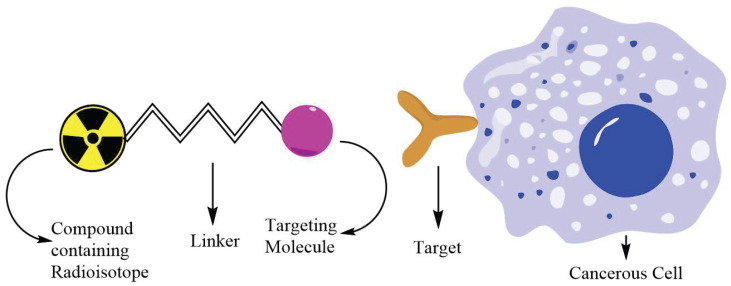
Graphical representation of Radiopharmaceuticals.

Radiopharmaceuticals entered clinical practice around the 1930s [19]; currently, many radionuclides have been identified and some compounds based on them have been developed with specific biological activity towards various types of cancer [20]. Radiopharmaceuticals are biologically active molecules labeled by radionuclides which provide a beneficial source of ionizing radiation mainly applied in diagnostic imaging and therapy [21]. Radiopharmaceutical therapy (RPT) involves the delivery of radionuclides for use in the treatment of patients with cancer [22]. RPT radionuclides emit α or β particles (helium nuclei or electron, respectively), or γ rays (very high energy photons) depending on their specific radioactive decay mechanism, or can produce high energy Auger electrons [22]. Similar to external beam radiation, each emission type can cause damage to cellular DNA and organelles, and generate destructive radical oxygen species, all of which can result in cell death [22,23]. Radiopharmaceuticals generally consist of a molecule that contains at least one radioisotope, a targeting molecule that can specifically recognize and bind to cancer cells, and a link between these two elements, as depicted in Figure 1; but it can also refer to a radionulcide salt that is capable of localize to specific tissues because of their biomolecular properties [24], for example, ^89^Sr naturally localizes to bone tissues [25], and ^131^I accumulates in thyroid tissues. Other radionuclides need a labeling compound in order to reach the desired target.

This review aims to provide a comprehensive analysis of the current approaches and future directions in the use of therapeutic radiopharmaceuticals for the treatment of pancreatic cancer, referring predominantly to PDAC and exploring the evolving landscape of radiopharmaceutical therapy, highlighting the potential benefits, limitations, and emerging trends in the field.

Through an examination of established techniques and newer radiopharmaceutical technologies, the goal is to shed light on the transformative impact of these agents in pancreatic cancer therapy and to underscore the importance of further research to optimize efficacy, address challenges, and unlock the full spectrum of innovative strategies aimed at enhancing patient outcomes in this complex oncological scenario. Furthermore, the most recent clinical trials in the field of treatment of pancreas cancer are presented. This review, hence, aims to bring to the reader’s attention what modern radiological methods are and to present an updated library of (radio)molecular systems used to treat one of the most aggressive forms of cancer.

## 2. Understanding Pancreatic Cancer and Its Challenges

### 2.1. Pathogenesis and Genetic Alterations

The low survival rates associated with PDAC primarily reflect the fact that tumors progress rapidly with few specific symptoms and are thus at an advanced stage at diagnosis, with only 10% being operable [26]. PDAC accounts for more than 90% of all pancreatic tumors, which constitute a heterogeneous group of diseases encompassing cancers of the endocrine and exocrine pancreas [26].

The transformation of a normal cell into a malign one involves various mutations and epigenetic modifications [27]. Genetic studies imply that PDAC arises from one of three known precursor lesions, including pancreatic intraepithelial neoplasias (PanINs), intraductal papillary mucinous neoplasms, and mucinous cystic neoplasms. The majority of PDAC cases develop from PanINs, progressing from PanIN1A and -1B to PanIN-3 [26,28]. NGS (next generation genome sequencing) and computational biology have provided a comprehensive understanding of the genetic alterations driving the genesis and progression of pancreatic cancer. The most common genetic alterations in pancreatic cancer include KRAS (Kirsten rat sarcoma virus) mutations, which are present in approximately 90% of cases [29]. Among these, mutations like G12D, G12V, and G12C are the most prevalent. KRAS mutations impair GTPase activity, affecting various signaling pathways and serving as a marker for poor prognosis. However, other genes like TP53, CDKN2A, and SMAD4 also play crucial roles in pancreatic cancer development [30]. Epigenetic alterations involving genes regulating chromatin remodeling and histone modifications are frequently observed in pancreatic tumorigenesis [29].

Histologically, PDAC is characterized by a dense stromal matrix that consists of various cellular and acellular components [31]. This stromal matrix is also referred to as a desmoplastic reaction or tumor microenvironment (TME) and starts to evolve early around PanIN lesions [31]. In addition, the pancreatic TME has an abundant fibrotic stroma, rich in various cell types and extracellular components including collagen, fibronectin, and hyaluronic acid [32]. Cells within the tumor microenvironment (TME) interact with each other and produce various components such as ECM (extracellular matrix, growth factors (e.g., TGF, VEGF), matricellular proteins (e.g., SPARC, tenascin), enzymes (e.g., MMPs, TIMPs), and cytokines, creating a complex signaling network with tumor cells [31,33]. Single-cell RNA sequencing studies on human pancreatic adenocarcinoma (PDAC) samples have revealed a heterogeneous mixture of cancer cell subtypes, distinct fibroblast and macrophage subpopulations, and significant alterations in the TME post-chemotherapy. These studies highlight the complexity of the PDAC TME and its response to therapy [31,33].

### 2.2. Metabolic Dysregulations

Pancreatic cancer induces metabolic dysregulation, which holds significant importance in the pathology of the disease. This dysregulation encompasses alterations in glucose metabolism, amino acid metabolism, and lipid metabolism, contributing to the progression and characteristics of pancreatic cancer cells [34].

Among what was reported before, the main challenges to face while dealing with pancreatic cancer are as follows: (1) hypoxic microenvironment—highly hypoxic conditions in pancreatic tumors upregulate glycolytic activity through HIF-1α, contributing to metabolic adaptations that sustain cancer cell survival [35]; (2) therapeutic resistance—alterations in metabolic pathways, such as increased fatty acid synthesis, can lead to resistance to conventional treatments like gemcitabine [36]; (3) metabolic plasticity—pancreatic cancer cells exhibit compensatory metabolic networks that allow them to adapt to changes in nutrient availability, posing challenges for targeted therapies [29].

### 2.3. Screening and Diagnosis Techniques

Another critical point concerning PC is the screening and diagnosis techniques. PC is currently not suitable for population-based screening due to its low incidence and the lack of accurate, affordable, and non-invasive screening tests, leading to late-stage diagnosis with symptoms often indicating advanced disease [37]. As such, only 10–20% of newly diagnosed PDAC patients are eligible for a potentially upfront curative resection [38].

The methods currently used for PC diagnosis mainly concern imaging (computed tomography, magnetic resonance imaging, endoscopic ultrasound, and others), serological tests (carbohydrate antigen 19-9, carcinoembryonic antigen, carbohydrate antigen 125, carbohydrate antigen 242, macrophage inhibitory cytokine-1, mucin 5AC), liquid biopsies (circulating tumor DNA, circulating tumor cells, exosomes, microRNAs) [39]. More precisely, Multidetector computed tomography (MDCT) with angiography, performed during the pancreatic arterial (40–50 s) and portal venous (65–70 s) phases, has emerged as the gold standard imaging modality for pancreatic cancer evaluation worldwide. This technique yields high-resolution 3D multiplanar reconstructions, facilitating precise assessment of tumor dimensions, local invasion, vascular involvement, and metastatic spread [40,41,42,43].

Future advancements in cancer biomarkers and ’omics’ technologies for early detection of pancreatic cancer include: metabolomics, whose studies aim to identify metabolic changes associated with pancreatic cancer, offering insights into potential biomarkers (which are defined characteristics that can be measured as an indicator of normal biological processes, pathogenic processes or responses to an exposure or intervention for early detection [44]) for early detections; saliva analysis—saliva metabolome, transcriptome, and microbiome studies; a fecal microbiota signature with high specificity for pancreatic cancer; proteomic and glycomic biomarkers; machine learning applications in precision oncology and ultrasensitive fluorescence nanobiosensors [37].

## 3. The Role of Radiopharmaceuticals in the Treatment of Cancer

### 3.1. Overview of Radiopharmaceuticals

As reported before in this review, radiopharmaceuticals are active compounds, composed of a radionuclide (responsible for the ionizing radiation) and a targeting compound capable of carrying or delivering the radioactive nuclide to the desired location, the targeted organ, tissue or lesion [45]. Often, these two elements are connected by a linker.

Tumors and cancer cells undergo exposure to ionizing radiation, a process that damages the DNA of the cells, thereby impeding their growth and proliferation [46]. Generally, there are two primary methods for subjecting tumors to ionizing radiation. Firstly, tumors can be irradiated using an external radiation source, such as an orthovoltage X-ray machine, a linear accelerator, a Cobalt-60 source, a neutron beam generator, or a proton beam generator [46]. Secondly, tumors can be subjected to a radioisotope, either through implantation directly into the tumor or through systematic administration, leading to the continuous emission of ionizing radiation over time [46,47,48].

### 3.2. Classification and Applications of Radiopharmaceuticals

**Figure 2:**
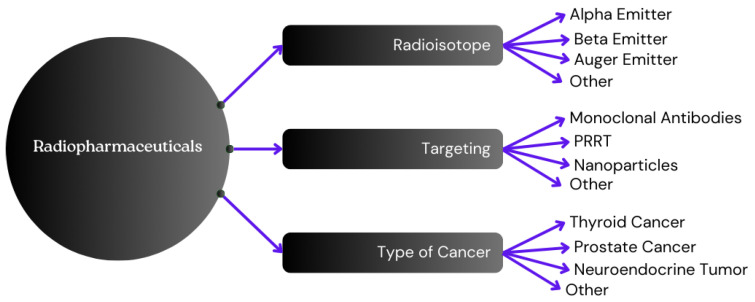
Schematic representation of Radiopharmaceuticals categorization (PRRT stands for Peptide Receptor Radionuclide Therapy).

This subsection discusses the classification of radiopharmaceuticals based on their types, mechanisms of action, and clinical considerations. A schematic representation is depicted in Figure 2.

#### 3.2.1. Types of Radioisotopes

Radiopharmaceuticals can be divided into various types based on their specific activity and applications and several classifications are possible. A crucial aspect of radiopharmaceutical classification is the type of radioisotope they contain. These radioisotopes emit different kinds of radiation, such as X-rays, positrons, γ rays, or β particles (electron or positron), each with unique properties and clinical applications. For molecular imaging, since the aim is to detect possible cancerous tissues, it is preferable to use radionuclides that are rather penetrating but not quite ionizing (γ or $\beta^{+}$—emitting radionuclides). Radionuclides used for therapy are lower penetrating but more energetic, hence, they havemore ionizing emissions ($\beta^{-}$, α or Auger $e^{-}$ emitters). For example, γ emitters like ^99^Tc are commonly used in diagnostic imaging due to their favorable imaging characteristics and low radiation dose to patients [49,50]. Positron emitters like ^18^F are utilized in positron emission tomography (PET) imaging, enabling high-resolution imaging of metabolic processes in the body [51]. β emitters such as ^177^Lu are employed in targeted radionuclide therapy for various cancers, including neuroendocrine tumors and metastatic prostate cancer. Lu177-tagged radioligands are molecules precisely designed to target and bind to specific receptors or proteins characteristic of targeted cancer [52]. $\beta^{-}\;\mathrm{particles}$ particles can penetrate large volumes of tissue, making them suitable for treating extensive tumors [50]. However, their long range also means that surrounding, untargeted cells may receive radiation exposure, known as the cross-fire effect. In contrast, α particles have a shorter range and are effective for targeting smaller tumors, particularly in hematological cases, through targeted α-therapy. Auger electrons, which irradiate at subcellular levels, are particularly effective for delivering high radiation doses directly to cancer cell nuclei, minimizing damage to adjacent healthy tissue. The main challenge with Auger electrons lies in ensuring precise targeting of the intended site [50,53,54,55]. Table 1 reports the most common radioisotopes used in therapy.

#### 3.2.2. Mechanisms of Action

Radiopharmaceuticals can be classified based on their mechanism of action and targeting specificity. Targeted radiopharmaceuticals are a specialized category of radiopharmaceuticals designed to deliver radiation specifically to target cells or tissues, such as cancer cells while minimizing exposure to healthy tissues. These radiopharmaceuticals utilize various targeting mechanisms to enhance their specificity and efficacy in cancer therapy. One important example is monoclonal antibodies, which are commonly used in targeted radiopharmaceutical therapy. They are designed to specifically bind to antigens expressed on the surface of cancer cells. By attaching a radionuclide to the monoclonal antibody, the radiation can be delivered directly to the cancer cells, leading to localized cell destruction [56].

Peptide receptor radionuclide therapy (PRRT) is a type of targeted radionuclide therapy that involves the systemic administration of therapeutic peptides labeled with radionuclides that selectively target cancer cells [57]. Radiolabeled SSAs (Somatostatin analogs) are the preferred choice for PRRT, as the receptor–peptide complex is internalized via endocytosis and the radionuclide is preferentially retained by the receptor-expressing tumor cells [57,58].

Another promising targeting strategy is provided by nanoparticle-based radiopharmaceuticals, where these radiopharmaceutical delivery systems utilize nanoparticles to improve the efficacy of radiopharmaceuticals. Nanoparticles, due to their unique properties such as size and surface characteristics, can enhance drug availability, reduce toxicity, and increase efficiency [59].

**Figure 3:**
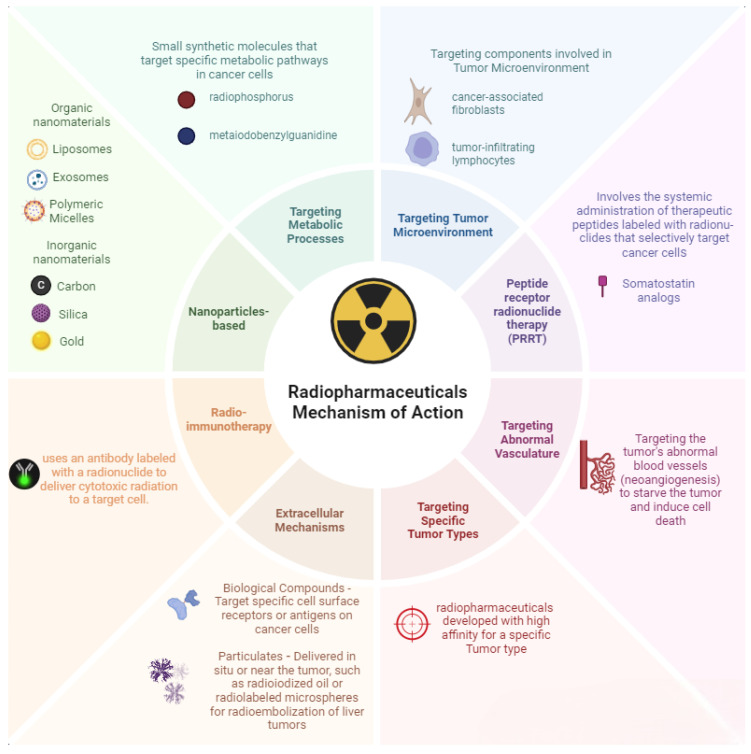
Illustration depicting various mechanisms of actions of radiopharmaceuticals [50,60].

Summarily, the classification of radiopharmaceuticals encompasses various types based on their specific radioisotopes, applications, and targeting mechanisms, with a focus on distinguishing between diagnostic and therapeutic radiopharmaceuticals, as well as considering the type of radiation emitted and the mode of action, such as targeted versus non-targeted delivery approaches. A summary and schematic illustration of the different mechanisms of actions of radiopharmaceuticals is reported in Figure 3.

The decision regarding which radiopharmaceutical to use is predominantly driven by the specific characteristics and requirements of the cancer under consideration. Different types of cancers exhibit unique biological behaviors, molecular signatures, and therapeutic vulnerabilities. Therefore, the choice of radiopharmaceutical is carefully tailored to match the distinct features of each cancer type [61].

The first radiopharmaceutical approved by the FDA is radioiodine I131 [62], which is used specifically for thyroid cancer [63]. Differentiated thyroid cancer metastases retain the ability to concentrate iodine for thyroglobulin hormone production. High concentrations of radioiodine (^131^I) may be delivered via this mechanism leading to tumor cell death by beta particle irradiation [64].

In the realm of cancer therapy, RPT presents a unique blend of attributes from both chemotherapy and targeted biological therapy [64]. While chemotherapy primarily inhibits signaling pathways associated with malignancy, RPT distinguishes itself by its ability to kill cancer cells [64,65]. However, unlike chemotherapy, RPT targets cells based on the expression of tumor-specific markers rather than their rate of proliferation [64].

Over the past few years, the repertoire of available or investigational RPTs has steadily expanded. The FDA approval and successful market penetration of ^223^RaCl_2_ (Xofigo™), an α-emitter used for the treatment of patients suffering from castration-resistant prostate cancer with bone metastases and no visceral metastases [66], have underscored the viability of α-emitter-based RPTs (αRPT) [64,67]. α particles are potent ionizing agents classified as high Linear Energy Transfer (LET) radiation [68]. Since α particles cannot be directly visualized in vivo, the gamma photons, characteristic X-rays, or bremsstrahlung radiation emitted during the decay of the parent radionuclide are commonly utilized for quantifying target uptake, conducting dosimetry assessments, and evaluating therapy response [68]. Quantifying target uptake, dosimetry, and therapy response. Complex molecular pathways are initiated when α-particles interact with biological tissue [68,69]. High Linear Energy Transfer (LET) radiation primarily targets DNA, and even a single α particle track can cause irreparable double-strand breaks [68,69,70]. The passage of α tracks through the nucleus is associated with cytotoxicity, while traversal through the cytoplasm leads to less severe radiation-induced effects [68,69,70,71]. Conversely, β particle irradiation predominantly induces single-strand breaks, demonstrating approximately 500 times lower cytotoxic potency compared to α particles [68].

While RPTs utilizing β particle emitters (such as ^131^I131I, ^177^Lu, and ^90^Y) have amassed considerable clinical experience, much of it has been gleaned outside of rigorous clinical trials, making their comparative efficacy challenging to ascertain [64]. Nonetheless, the observed positive clinical outcomes, particularly in cases where other treatment options have been exhausted, have spurred pharmaceutical companies to initiate FDA registration trials, aiming for the commercialization of these agents [64].

A notable example of this trend is the recent FDA approval of Lutathera™, a ^177^Lu-labeled anti-somatostatin peptide RPT, for the treatment of somatostatin receptor-positive gastroenteropancreatic neuroendocrine tumors [64,72].

Clinically approved radiopharmaceuticals utilizing Y90 have been effectively employed in treating diverse cancer types. These formulations involve the coupling of Y90 chelators, such as DTPA (diethylene-triamine-pentaacetate) or DOTA (2,$2^{\prime \prime }$,$2^{\prime \prime }$,$2^{\prime \prime \prime }$-(1,4,7,10-Tetraazacyclo-dodecane-1,4,7,10-tetrayl) tetraacetic acid), with cell receptor-targeting motifs, such as peptides or antibodies, facilitating the targeted delivery of therapy to cancerous cells. For instance, the conjugation of DTPA or DOTA to various peptides enables the precise localization of chelate ions to distinct anatomical sites within the body [73].

Unlike α and β radiation, Auger electron emitter-based therapy necessitates the precise targeting of the radioisotope to individual tumor cells, including their nuclei [74]. The optimal efficacy of Auger radiation occurs when the emitters are firmly bound to DNA [74]. Despite encountering numerous challenges, Auger radiation therapy strategies continue to hold promise due to their selective toxicity towards cells incorporating the radiopharmaceutical into their nuclei [74]. In contrast to alpha and beta-minus radiation, Auger radiation emitters exhibit low toxicity when circulating in the blood or bone marrow but demonstrate heightened efficiency upon integration into the DNA of target cells [74].

## 4. Radiopharmaceuticals Therapy for Pancreatic Cancer

### 4.1. Clinical Management of Pancreatic Cancer

At the clinical level, treatment selection is primarily guided by an analysis of the disease stage, with particular consideration given to the presence or absence of metastases, and thus, its spread to adjacent organs, nerves, and blood vessels [75]. Pancreatic resection surgery represents the most effective therapeutic option in cases where tumor removal is feasible, namely when metastases have not spread to distant sites within the abdominal cavity, liver, or lungs, or when there is no extensive involvement of major nearby blood vessels [75].

For non-surgically treatable, yet non-metastatic forms, chemotherapy is administered to shrink the tumor size to render it operable. Additionally, in patients already identified as candidates for surgery, preoperative chemotherapy may be administered to eliminate tumor cells that may have spread beyond the pancreas.

In instances where disease extension at the time of diagnosis precludes surgical intervention, radiation therapy may be employed, either in conjunction with or independently of chemotherapy, to minimize local disease extension and organ-dependent symptoms. Furthermore, postoperative radiation therapy is advised in cases of extensive nodal involvement or inadequate resection as determined by definitive histological examination [75,76,77].

### 4.2. Current Radiopharmaceuticals

The treatment of pancreatic cancer remains challenging despite recent advances in radiopharmaceuticals. While various radioisotopes and radiopharmaceuticals have been explored for different types of cancer, the efficacy in pancreatic cancer has been limited.

#### Radiopharmaceuticals for NETs Treatment

**Figure 4:**
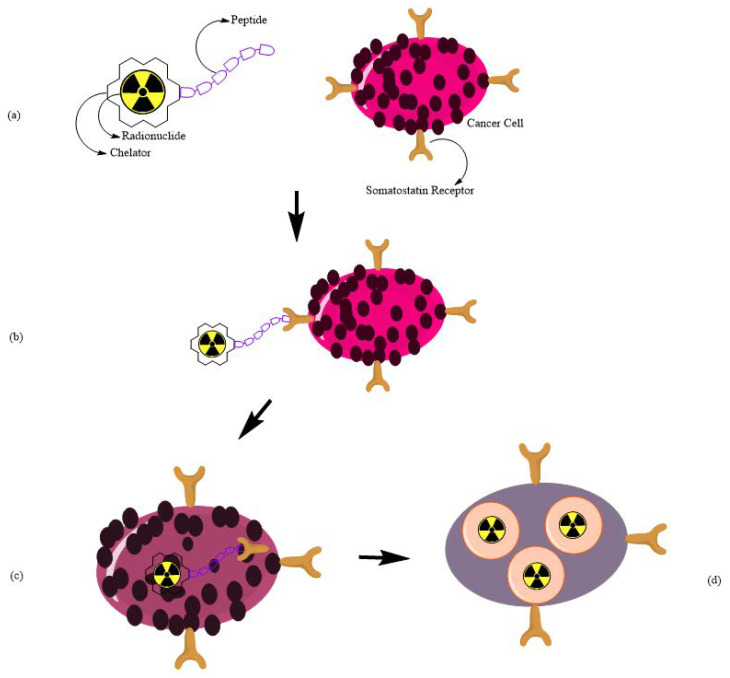
Schematic representation of Peptide Receptor Radionuclide Therapy (PRRT) mechanism; (**a**) on the left, radiopharmaceutical composed of radionuclide, chelator and a peptide; on the right a generic cancer cell exhibiting a somatostatin receptor; (**b**) binding between the radiopharmaceutical and the somatostatin receptor; (**c**) internalization of the radiopharmaceutical in the cancerous cell; (**d**) radioactivity of the radionuclide in the target cell.

As reported before, one promising therapy concerns Peptide Receptor Radionuclide Therapy (PRRT), which is an advanced form of molecular radiotherapy designed to target specific receptor molecules on cancer cells, notably NETs, delivering a precise and effective dose of radiation therapy. Known for its efficacy, low toxicity profile, and convenient treatment schedule, PRRT has rapidly emerged as a highly promising and evolving therapeutic approach in cancer care [78]. A schematic representation of the PRRT mechanism is shown in Figure 4.

**Figure 5:**
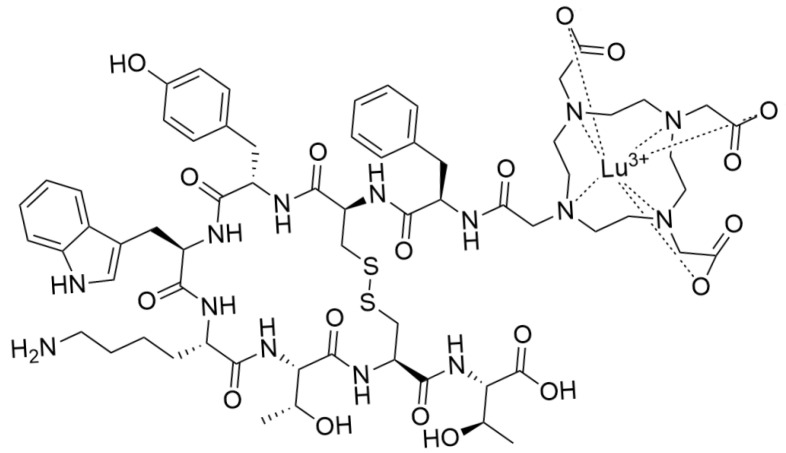
Structure of Lutathera^®^ (Lu177).

A notable radiopharmaceutical approved in North America and Europe showing promise in managing neuroendocrine tumors (NETs) is ^177^Lu DOTATATE, a peptide receptor radionuclide therapy (PRRT) agent targeting somatostatin receptors (SSTRs) commonly overexpressed in these tumors [79]. Among all other chelator-peptide analogs (TETA, TEMA, SAR) DOTATATE is the most studied and clinically used (tagged with Lu177/Y90) worldwide [78] (Figure 5 shows the chemical structure of Lutathera^®^). PRRT comprises binding a radiolabeled hormone analogous to SSTRs on tumor cells, quickly clearing leftover radioactivity, and long-term retention of radioactivity in the cells. PRRT outperforms external beam irradiation and systemic chemotherapy by targeting tumor cells more effectively and with fewer adverse effects [80].

According to the joint IAEA, EANM, and SNMMI practical guidance on peptide receptor radionuclide therapy in neuroendocrine tumors [81], PRRT is indicated for the treatment of patients with positive expression of SSTR2 (which is the most frequently expressed subtypes in both gastroenteropancreatic NETs and also in normal tissue [82]), or metastatic or inoperable NETs. PRRT can also be considered as first-line in patients with extensive metastatic/large-bulk disease at diagnosis [83].

Studies predominantly focus on Yttrium (Y90) and Lutetium (Lu177) isotopes, showing PRRT’s efficacy in gastroenteropancreatic NETs with response rates of 15–40%. PRRT offers significant benefits, including improved disease control and prolonged progression-free periods [80,84,85,86]; it is considered to be a very well-tolerated therapy by patients, with very few and mild side effects if adequate precautions are taken [78]. However, questions remain regarding optimal sequencing, patient selection, and combination therapy [80,84,85,86].

## 5. Novel Approaches in Radiopharmaceutical Therapy for Pancreatic Cancer

### 5.1. Radioimmunotherapy

In recent years, radioimmunotherapy has emerged as a promising approach for the targeted treatment of PDAC. Radioimmunotherapy works by utilizing radiolabeled antibodies that specifically target antigens overexpressed on the surface of pancreatic cancer cells. These antibodies deliver radiation directly to the cancer cells, inducing DNA damage and cell death; additionally, the radiation emitted by the radionuclide can have a bystander effect (which refers to the radiation effects induced in non-irradiated cells as a result of cellular signaling from nearby, irradiated cells [87]), further enhancing the treatment efficacy by affecting neighboring cancer cells [88].

Several targets can be selected with radioimmunotherapy (RIT). In general, an ideal RIT target should be selected based on its expression profile on cancerous and normal cells, low blood circulation, and high affinity to the intended radioimmunoconjugate [88].

One of these targets has been investigated by Jiao et al. They found that Centrin1 (CETN1) and Centrin2 (CETN2), which are multi-functional calcium-binding phosphoproteins overexpressed in PDAC, can be addressed by specific antibodies; when they are radiolabeled with 213 Bi, they show effectiveness in radioimmunotherapy of PDAC xenografts, providing a safe and CETN1-specific treatment option [89].

Aung et al. investigated the role of α6β4 integrin in PC and demonstrated that it is a good target employing single-photon emission computed tomography (SPECT) or near-infrared (NIR) imaging for immunotargeting [90]. The overexpression of α6β4 is observed in the early stages of pancreatic adenocarcinoma termed pancreatic intraepithelial neoplasias (PanINs) and holds great promise as an early biomarker [90,91]. The authors isolated a fully human monoclonal immunoglobulin (Ig) G1 antibody against α6β4, designated as ITGA6B4, from a large-scale human antibody library constructed using a phage-display system and screened using living pancreatic cancer cells, and they labeled ITGA6B4 with Indium-111 (^111^In) for single-photon emission computed tomography (SPECT) and near-infrared (NIR) fluorophore indocyanine green (ICG) for NIR fluorescence imaging [90,92].

Hausner et al. performed the first-in-human study aimed to evaluate the safety, pharmacokinetics, and imaging capabilities of the peptide in patients diagnosed with metastatic cancers, specifically lung, colon, breast, and pancreatic cancers [93]. The authors developed ${[}^{18}F]\alpha v\beta 6$-Binding Peptide as a novel PET imaging agent for a broad spectrum of malignancies. The key findings of this study are as follows: the safety and tolerability of ${[}^{18}F]\alpha v\beta 6$-Binding Peptide as it was well tolerated by the participants with no significant adverse side effects noted. PET images showed the substantial uptake of ${[}^{18}F]\alpha v\beta 6$-Binding Peptide in both primary tumors and metastatic locations, such as the brain, bone, liver, and lung. This indicates that the peptide effectively targets the integrin, which is overexpressed in several epithelial-derived cancers, offering a dependable approach for detecting malignancies [93].

Another RIT proposal has been studied by Yoshii et al. They demonstrated that adjuvant Cu64-RIT with ^64^Cu-PCTAcetuximab (where PCTA is the chelator acronym), following the surgical resection of PC, significantly prolongs survival, while exhibiting minimal toxicity [94]. In comparison to conventional adjuvant chemotherapy with gemcitabine in a mouse orthotopic xenograft model, this therapy shows an overall better outcome. Notably, it effectively suppresses local recurrence, hepatic metastasis, and peritoneal dissemination, which are typically associated with the poor prognosis of PC patients. These findings suggest that adjuvant Cu64-ipRIT with Cu64-PCTA-cetuximab represents a promising novel treatment option to enhance the condition of PC patients post-surgery [94].

RIT seems to be a promising approach to PDAC, but optimizing it presents a persistent challenge, especially in addressing advanced stages of the disease [88].

### 5.2. Nanotechnologies

Extensive research has been conducted leveraging the principles of nanotechnology for therapeutic drug delivery [95]. While each system possesses unique characteristics, the objective in cancer therapy is to achieve improved drug absorption and permeability, alongside site specificity and control over drug release rate [95,96].

Nanoparticles exhibit the capability to (a) passively target tumor vasculature via the enhanced permeability and retention effect (EPR) [95,97], or (b) actively target specific sites by functionalizing them with site-specific ligands [98]. The conjugation of nanoparticles with cytotoxic agents can augment tumor penetration, leading to more effective treatments [95,99,100,101].

Trujillo-Nolasco et al. synthesized a new nanosystem based on the use of targeted therapy and radiotherapy to produce a synergistic effect [102]. PC often involves the activation of the KRAS oncogene (Kirsten rat sarcoma 2 viral oncogene homolog) during tumor formation. The novel therapeutic approach proposed by Trujillo-Nolasco et al. involves the use of the C19 molecule ((2S)-N-(2,5-dichlorophenyl)-2-[(3,4-dimethoxyphenyl)-methylamine]propanamide), which disrupts KRAS-membrane association in cancer cells. Additionally, pancreatic cancer commonly exhibits the overexpression of the chemokine receptor CXCR4 [102,103]. This research proposes a dendrimer-based nanoradiopharmaceutical (177Lu-DN(C19)-CXCR4L) that encapsulates C19 and targets CXCR4 receptors, serving as both a targeted radiotherapy system using lutetium-177 and an oncotherapeutic agent by stabilizing the KRAS4b-PDE complex, thus offering a dual-specific therapy for pancreatic cancer. The synthesized nanosystem demonstrated efficient encapsulation of C19, a favorable particle size, and specific uptake in pancreatic cell lines [102]. Significant cytotoxic effects were observed, particularly in the KRAS-dependent and radioresistant cell line Mia PaCa-2, which expresses high levels of CXCR4 receptors. Treatment with a radiation dose of 3 Gy/Bq, attributed to the presence of C19, significantly decreased cell viability, showcasing a synergistic effect between radiotherapy and chemotherapy in inducing apoptosis in pancreatic cancer cells [102].

Gold nanoparticles (AuNPs) offer promising medical applications. This is due to their radioactive isotopes such as 198Au and 199Au. 198Au, with its 2.70-day half-life, emits β (961 keV) and γ radiation (411 keV γ (96%) [104]. This enables efficient destruction of tumor cells and tissue. The ideal beta penetration range and suitable half-life of 198Au provide crossfire effects to destroy tumor cells and tissue while minimizing radiation exposure to neighboring non-target tissues [104]. A key advantage of radioactive gold nanoparticles is their ability to exert therapeutic effects even when taken up by only a subpopulation of tumor cells. This is due to the path length of the emitted radiation. 198Au also allows for monitoring therapeutic response through scintigraphic imaging, facilitating the evaluation of tumor retention characteristics and pharmacokinetics [104]. Recently, radioactive analogs of mangiferin-functionalized gold nanoparticles (MGF-AuNPs) have been synthesized and characterized. Mangiferin (a compound extracted from mango fruit [105]) displays tumor cell avidity with high selectivity toward receptors over-expressed by a host of tumors that includes PC [104,106,107,108].

### 5.3. Theranostic Approaches

Theranostics is a concept that represents the combination of therapy and diagnostics, and, more precisely, it refers to the use of RPTs containing radionuclides for imaging (hence for the diagnosis of the disease) and therapy [109,110]. Watabe et al. developed a novel theranostic approach using radioactive monoclonal antibodies (mAbs) targeting glypican-1 (GPC1), a protein overexpressed in PDAC tumors. The diagnostic is provided by Zr89 labeled with mAbs; through Pet scanning, it is possible to localize the tumoral tissue due to the high uptake in GPC1-expressing cells [111]. The same mAbs are tagged with radioactive At211 for targeted α therapy. This treatment causes DNA damage in cancer cells, leading to a marked reduction in tumor growth in experimental modes. Nanoparticles have also been designed to deliver imaging agents in conjunction with chemotherapeutic therapeutic agents [112]. An example of this approach is the work of Wang et al. that engineered cetuximab-modified gemcitabine-loaded magnetic albumin nanospheres to enhance targeted therapy and imaging for pancreatic cancer cells. This strategy showed improved therapeutic effectiveness by utilizing thermochemotherapy combined with magnetic targeting techniques [113]. The capability to use a single species or molecular entity for both diagnosis and therapy at the same time is extremely promising and may offer new medical avenues for cancer treatment, as evidenced by the success of agents like Ga68Lu177-DOTATATE in NETs [114]. Its promising efficacy has facilitated its progression to first-in-human clinical trials, which are currently underway for the treatment of PDAC [115]. The conclusions of these initial clinical trials are that ${[}^{68}\mathrm{Ga}]\mathrm{Ga}$ DOTA-5GPET/CT can be used properly for the diagnosis and thus the selection of patients for treatment and that ${[}^{177}\mathrm{Lu}]\mathrm{Lu}$ DOTA-ABM-5G is able to reach the metastasis and be retained and that the theranostic combination of the two elements is safe and well tolerated [115]. A challenging aspect of the theranostic approaches related to PDAC is the paucity of specific molecular targets and the characteristic dense stromal barrier of these tumors. It is crucial to identify novel and specific molecular targets.

### 5.4. Photothermal Therapy Combined with Radiopharmaceuticals

**Figure 6:**
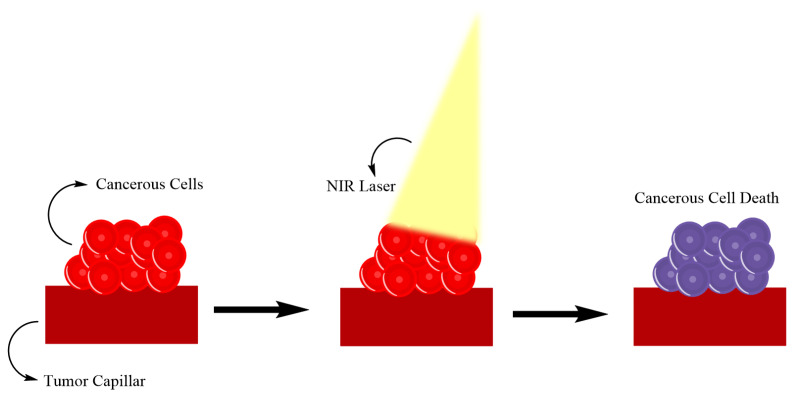
Phototermal Therapy mechanism scheme.

Photothermal therapy is a technique that uses photothermal effects induced by photothermal agents that convert light energy into heat, thus increasing the temperature of surrounding tissue and triggering cell death [116]; Figure 6 illustrates this mechanism. Shi et al. developed a semiconducting polymer nano-radiopharmaceutical labeled with therapeutic radioisotope Lu177 (177Lu-SPN-GIP) for combined radio- and photothermal therapy (PTT) [117]. This nano-radiopharmaceutical demonstrated good stability, high cell uptake, and long retention time in the tumor site. Combining radiotherapy and photothermal therapy, Lu177-SPN-GIP showed enhanced therapeutic capability in killing cancer cells and xenograft tumors in living mice compared to RT or PTT alone [117]. Additionally, it was found to suppress the growth of tumor stem cells and reverse epithelial–mesenchymal transition (EMT), potentially reducing the occurrence of metastasis [117].

An et al. developed a novel combination therapy strategy, termed RNT-SDT-PTT, which combines radionuclide therapy, sonodynamic therapy (which involves the sensitization of target tissues with a non-toxic sensitizing chemical agent and subsequent exposure of the sensitized tissues to relatively low-intensity ultrasound [118]), and photothermal therapy for the treatment of PC [119]. They utilized polydopamine-modified Au nanostars loaded with protoporphyrin (IX) and labeled with diagnostic (99mTc) or therapeutic (131I) radionuclides for precise diagnosis and treatment [119]. The intratumoral injection of 131I-AN@D/IX allowed for targeted delivery, maximizing local dose absorption and reducing the required number of injections [119]. The synergistic effects of the combined therapy were demonstrated to significantly inhibit tumor growth in pancreatic tumor-bearing mice [119]. The study highlighted the potential of this multi-modal therapy approach to enhance therapeutic efficacy and improve treatment outcomes.

A novel photothermal-based nanoparticle, composed of anti-urokinase plasminogen activator receptor (uPAR), polyethylene glycol (PEG), and indocyanine green-modified gold nanocapsules, has shown promising results in enhancing the median survival rate of complete ablation [120,121]. When combined with Iodine-125 (125I) interstitial brachytherapy (IBT-125I), this nanoparticle formulation improved the median survival rate by 25% with a single intervention [120,121].

### 5.5. Latest Clinical Trials

Antibody-based targets seem to be one of the most promising mechanisms to address PDAC and thus to be translated into clinical practice. Several antibody-based targets have been developed and primarily assessed in preclinical studies [122,123]. CA 19-9 is known to be the marker for PDAC. Different agents have proven to have a high affinity for CA 19-9, but in particular mAb, 5B1 [124]. Viola-Villegas et al. developed Zr89-5B1, a fully human, antibody-based radiotracer that targets tumor-associated CA 19-9 performing preclinical tests and demonstrating that Zr89-5B1 is able to detect orthotopic models of PDAC [125]. In 2019 Lohrmann et al. reported the first clinical trial of ${[}^{89}\mathrm{Zr}]$Zr-HuMab-5B1 [126]. It was demonstrated that this system can detect the primary sites of PDAC, metastases, and small lymph nodes [122,126].

A recent study aimed to apply static and dynamic PET/CT using Ga68-labeled Fibroblast Activated Protein Inhibitors (Ga68-FAPI-PET/CT) in 32 preoperative, treatment-naive patients with ambiguous pancreatic masses to assess the potential diagnostic utility of this novel imaging technique in distinguishing between mass-forming pancreatitis and PDAC [127].

The majority of the ongoing clinical trials concern NETs, and this confirms again that PDAC treatment is far from the resolution.

## 6. Challenges and Limitations

While radiopharmaceutical therapy holds significant importance in oncological treatments, there are crucial considerations that must not be overlooked.

One of the major issues associated with radiopharmaceutical therapy for the treatment of cancer in general is the resistance mechanisms of tumor cells [128,129]. Tumor cells develop resistance mechanisms against radiotherapy through various pathways. These include DNA damage repair, cell cycle arrest evasion, apoptosis escape, presence of cancer stem cells, alterations in cancer cell metabolism and microenvironment, as well as the involvement of exosomal and non-coding RNA, metabolic reprogramming, and ferroptosis [130]. Additionally, cancer cells exploit cytoprotective mechanisms like the p53-regulated pathways involving DNA repair enzymes, proteomic stress response, and antioxidant production to resist the cytotoxic effects of radiation and drugs [131]. Understanding these molecular alterations, such as DNA repair capacity, cell cycle checkpoints, and tumor metabolism, is crucial in overcoming radioresistance and improving the efficacy of radiotherapy [132]. Moreover, the use of X-ray responsive radiopharmaceutical molecules containing chemical radiosensitizers can enhance radiotherapeutic effects by inducing ROS levels and inhibiting DNA repair in cancer cells, thus sensitizing them to radiation [130].

Challenges for what concerns specifically PC include: the limited accuracy of conventional imaging modalities for tumor assessment, the need for improved tumor retention and uptake of radiotracers, and the difficulty in detecting and characterizing tumors using radiological imaging, especially post-neoadjuvant therapy [133]. Despite advancements in targeted radionuclide therapies, such as radiolabeled peptides and antibodies, pancreatic adenocarcinomas remain a significant challenge; therefore, continuous advancements in radiopharmaceutical development are crucial for improving the diagnosis and treatment outcomes of pancreatic cancer.

## 7. Conclusions

Pancreatic cancer is one of the deadliest of all of the solid malignancies. Despite the efforts that have been made to find proper therapeutic management of this kind of pathology, the final cure is still far.

To date, the European Medicines Agency (EMA) has not approved any radio drugs for treating pancreatic cancer. Even though some radiopharmaceuticals are allowed for different kinds of cancer like neuroendocrine tumors, the fact that there are no approved options for pancreatic cancer highlights the struggles in finding good treatments for this disease.

However, the development of targeted radiopharmaceuticals, such as radiolabeled antibodies and peptides, along with innovative nanotechnological approaches, holds promise for addressing these challenges.

Continued advancements in the development of radiopharmaceuticals, alongside a comprehensive understanding of tumor biology and resistance mechanisms, are indispensable for enhancing both the diagnosis and treatment outcomes of pancreatic cancer (PC). Through harnessing the potential of radiopharmaceuticals, significant progress can be made towards achieving more precise diagnoses, targeted therapeutic interventions, and ultimately, improved survival rates for patients not only with pancreatic cancer but also with various other malignancies.

## Figures and Tables

**Table 1:** Main radioisotopes used in therapy [50].

Radioisotope	Half-Life	Energy (MeV)	Tissue Penetration Range (mm)
*β-emitter*			
Y90	2.7 days	2.284	12
Tb161	6.9 days	0.593	3
Lu177	6.7 days	0.497	2.2
I131	8 days	0.81	2.4
Re188	17 h	2.118	11
*α-emitter*			
Tb149	4.1 h	3.97	<100 μm
At211	7.2 h	7.45	<100 μm
Pb212	10.6 h	8.78	<100 μm
Ra223	11.4 days	5.71, 6.82, 7.39, 6.62	<100 μm
Th227	18.7 days	6.14, 5.71, 6.82, 7.39, 6.62	<100 μm
Ac225	10 days	5.8, 6.3, 7.1, 8.38	<100 μm
*Auger $e^{-}$ emitter*			
I125	60 days	0.019	<1 μm
In111	2.8 days	0.007	<1 μm

## Data Availability

Not applicable.

## Abbreviations

The following abbreviations are used in this manuscript:

AuNPs	Gold Nanoparticles
CA 19-9	Carbohydrate antigen 19-9CA 19-9
CETN1	Centrin1
CETN2	Centrin2
DOTA	2,$2^{\prime }$,$2^{\prime \prime }$,$2^{\prime \prime \prime }$-(1,4,7,10-Tetraazacyclododecane-1,4,7,10-tetrayl)tetraacetic acid
DTPA	Diethylene-triamine-pentaacetate
EANM	European Association of Nuclear Medicine
ECM	Extracellular Matrix
EMT	Epithelial Mesenchymal Transition
EPR	Permeability and Retention Effect
GPC1	Glypican-1
IAEA	International Atomic Energy Agency
IBT	Interstitial Brachitherapy
ICG	Indocyanine Green
Ig	Immunoglobulin
KRAS	Kirsten Rat Sarcoma Virus
LAPC	Locally Advance Pancreatic Cancer
LET	Linear Energy Transfer
mAbs	Monoclonal Antibodies
MDCT	Multidetector Computed Tomography
MGF-AuNPs	Mangiferin-functionalized Gold Nanoparticles
NETs	Neuroendocrine Tumors
NGS	Next Generation Genome Sequencing
NIR	Near-Infrared
PanINs	Pancreatic Intraepithelial Neoplasias
PCTA	3,6,9,15-Tetraazabicyclo[9.3.1]pentadeca-1(15),11,13-triene-3,6,9-triacetic acid
PDAC	Pancreatic Ductal Adenocarcinoma
PEG	Polyethylene glycol
PET	Positron Emission Tomography
PRRT	Peptide Receptor Radionuclide Therapy
PTT	Phototermal Therapy
RPT	Radiopharmaceutical Therapy
SDT	Sonodynamic Therapy
SNMMI	Society of Nuclear Medicine and Molecular Imaging
SPECT	Single-Photon Emission Computed Tomography
SSAs	Somatostatin Analogs
TME	Tumor Microenvironment
uPAR	Urokinase Plasminogen Activator Receptor

## References

[1] Liu Z., Sun B., Xu A., Tang J., Zhang H., Gao J., Wang L. (2024). MICAL2 implies immunosuppressive features and acts as an independent and adverse prognostic biomarker in pancreatic cancer. Sci. Rep..

[2] Sung H., Ferlay J., Siegel R.L., Laversanne M., Soerjomataram I., Jemal A., Bray F. (2021). Global Cancer Statistics 2020: GLOBOCAN Estimates of Incidence and Mortality Worldwide for 36 Cancers in 185 Countries. CA Cancer J. Clin..

[3] Ferlay J., Partensky C., Bray F. (2016). More deaths from pancreatic cancer than breast cancer in the EU by 2017. Acta Oncol..

[4] Ilic I., Ilic M. (2022). International patterns in incidence and mortality trends of pancreatic cancer in the last three decades: A joinpoint regression analysis. World J. Gastroenterol..

[5] Goral V. (2015). Pancreatic Cancer: Pathogenesis and Diagnosis. Asian Pac. J. Cancer Prev..

[6] Wolfgang C.L., Herman J.M., Laheru D., Klein A.P., Erdek M.A., Fishman E.K., Hruban R.H. (2013). Recent progress in pancreatic cancer. CA Cancer J. Clin..

[7] Starzyńska T., Karczmarski J., Paziewska A., Kulecka M., Kuśnierz K., Żeber-Lubecka N., Ambrożkiewicz F., Mikula M., Kos-Kudła B., Ostrowski J. (2020). Differences between Well-Differentiated Neuroendocrine Tumors and Ductal Adenocarcinomas of the Pancreas Assessed by Multi-Omics Profiling. Int. J. Mol. Sci..

[8] Williamson L.M., Steel M., Grewal J.K., Thibodeau M.L., Zhao E.Y., Loree J.M., Yang K.C., Gorski S.M., Mungall A.J., Mungall K.L. (2019). Genomic characterization of a well-differentiated grade 3 pancreatic neuroendocrine tumor. Cold Spring Harb. Mol. Case Stud..

[9] Yoon K.-A., Woo S.M., Kim Y.-H., Kong S.-Y., Lee M.K., Han S.-S., Kim T.H., Lee W.J., Park S.J. (2019). Comprehensive cancer panel sequencing defines genetic diversity and changes in the mutational characteristics of pancreatic cancer patients receiving neoadjuvant treatment. Gut Liver.

[10] Roth M.T., Cardin D.B., Berlin J.D. (2020). Recent advances in the treatment of pancreatic cancer. F1000Research.

[11] Zhao Z., Liu W. (2020). Pancreatic Cancer: A Review of Risk Factors, Diagnosis, and Treatment. Technol. Cancer Res. Treat..

[12] Kolbeinsson H.M., Chandana S., Wright G.P., Chung M. (2023). Pancreatic Cancer: A Review of Current Treatment and Novel Therapies. J. Investig. Surg..

[13] Millikan K.W., Deziel D.J., Silverstein J.C., Kanjo T.M., Christein J.D., Doolas A., Prinz R.A. (1999). Prognostic factors associated with resectable adenocarcinoma of the head of the pancreas. Am Surg..

[14] Sohn T.A., Yeo C.J., Cameron J.L., Koniaris L., Kaushal S., Abrams R.A., Sauter P.K., Coleman J., Hruban R.H., Lillemoe K.D. (2000). Resected adenocarcinoma of the pancreas—616 patients: Results, outcomes, and prognostic indicators. J. Gastrointest. Surg..

[15] Raut C.P., Tseng J.F., Sun C.C., Wang H., Wolff R.A., Crane C.H., Hwang R., Vauthey J.-N., Abdalla E.K., Lee J.E. (2007). Impact of resection status on pattern of failure and survival after pancreaticoduodenectomy for pancreatic adenocarcinoma. Ann. Surg..

[16] Kamisawa T., Wood L.D., Itoi T., Takaori K. (2016). Pancreatic cancer. Lancet.

[17] Jiang S., Fagman J.B., Ma Y., Liu J., Vihav C., Engstrom C., Liu B., Chen C. (2022). A comprehensive review of pancreatic cancer and its therapeutic challenges. Aging.

[18] Ng S.P., Koay E.J. (2018). Current and emerging radiotherapy strategies for pancreatic adenocarcinoma: Stereotactic, intensity modulated and particle radiotherapy. Ann. Pancreat. Cancer.

[19] Brugarolas P., Comstock J., Dick D.W., Ellmer T., Engle J.W., Lapi S.E., Liang S.H., Parent E.E., Kishore Pillarsetty N.V., Selivanova S. (2020). Fifty Years of Radiopharmaceuticals. J. Nucl. Med. Technol..

[20] Shende P., Gandhi S. (2021). Current strategies of radiopharmaceuticals in theranostic applications. J. Drug Deliv. Sci. Technol..

[21] Crestoni M.E. (2018). Radiopharmaceuticals for Diagnosis and Therapy. Reference Module in Chemistry, Molecular Sciences and Chemical Engineering.

[22] Salerno K.E., Roy S., Ribaudo C., Fisher T., Patel R.B., Mena E., Escorcia F.E. (2023). A Primer on Radiopharmaceutical Therapy. Int. J. Radiat. Oncol. Biol. Phys..

[23] Abbott E.M., Falzone N., Lenzo N., Vallis K.A. (2021). Combining External Beam Radiation and Radionuclide Therapies: Rationale, Radiobiology, Results and Roadblocks. Clin. Oncol..

[24] Munjal A., Gupta N. (2024). Radiopharmaceuticals. [Updated 20 June 2023]. StatPearls [Internet].

[25] Battal H., Erdoğan S. (2023). The Role of Radiopharmaceuticals in the Bone Metastases Therapy. FABAD J. Pharm. Sci..

[26] Hidalgo M., Cascinu S., Kleeff J., Labianca R., Löhr J.-M., Neoptolemos J., Real F.X., Van Laethem J.-L., Heinemann V. (2015). Addressing the challenges of pancreatic cancer: Future directions for improving outcomes. Pancreatology.

[27] Kaur S., Kumar S., Momi N., Sasson A.R., Batra S.K. (2013). Mucins in pancreatic cancer and its microenvironment. Nat. Rev. Gastroenterol. Hepatol..

[28] Hruban R.H., Maitra A., Kern S.E., Goggins M. (2007). Precursors to pancreatic cancer. Gastroenterol. Clin. N. Am..

[29] Wang S., Zheng Y., Yang F., Zhu L., Zhu X.-Q., Wang Z.-F., Wu X.-L., Zhou C.-H., Yan J.-Y., Hu B.-Y. (2021). The molecular biology of pancreatic adenocarcinoma: Translational challenges and clinical perspectives. Sig. Transduct. Target. Ther..

[30] Takai E., Yachida S. (2015). Genomic alterations in pancreatic cancer and their relevance to therapy. World J. Gastrointest. Oncol..

[31] Hessmann E., Buchholz S.M., Demir I.E., Singh S.K., Gress T.M., Ellenrieder V., Neesse A. (2020). Microenvironmental Determinants of Pancreatic Cancer. Physiol. Rev..

[32] Stanciu S., Ionita-Radu F., Stefani C., Miricescu D., Stanescu-Spinu I.-I., Greabu M., Ripszky Totan A., Jinga M. (2022). Targeting PI3K/AKT/mTOR Signaling Pathway in Pancreatic Cancer: From Molecular to Clinical Aspects. Int. J. Mol. Sci..

[33] Ligorio M., Sil S., Malagon-Lopez J., Nieman L.T., Misale S., Di Pilato M., Ebright R.Y., Karabacak M.N., Kulkarni A.S., Liu A. (2019). Stromal Microenvironment Shapes the Intratumoral Architecture of Pancreatic Cancer. Cell.

[34] Sousa C.M., Kimmelman A.C. (2014). The complex landscape of pancreatic cancer metabolism. Carcinogenesis.

[35] Infantino V., Santarsiero A., Convertini P., Todisco S., Iacobazzi V. (2021). Cancer Cell Metabolism in Hypoxia: Role of HIF-1 as Key Regulator and Therapeutic Target. Int. J. Mol. Sci..

[36] Wang Z., Wang Y., Li Z., Xue W., Hu S., Kong X. (2023). Lipid Metabolism as a Target for Cancer Drug Resistance: Progress and Prospects. Front. Pharmacol..

[37] Søreide K., Ismail W., Roalsø M., Ghotbi J., Zaharia C. (2023). Early Diagnosis of Pancreatic Cancer: Clinical Premonitions, Timely Precursor Detection and Increased Curative-Intent Surgery. Cancer Control.

[38] Daamen L.A., Molenaar I.Q., Groot V.P. (2023). Recent Advances and Future Challenges in Pancreatic Cancer Care: Early Detection, Liquid Biopsies, Precision Medicine and Artificial Intelligence. J. Clin. Med..

[39] Yang J., Xu R., Wang C., Qiu J., Ren B., You L. (2021). Early screening and diagnosis strategies of pancreatic cancer: A comprehensive review. Cancer Commun..

[40] Martin-Perez E., Domínguez-Muñoz J.E., Botella-Romero F., Cerezo L., Matute Teresa F., Serrano T., Vera R. (2020). Multidisciplinary consensus statement on the clinical management of patients with pancreatic cancer. Clin. Transl. Oncol..

[41] Hidalgo M. (2010). Pancreatic cancer. N. Engl. J. Med..

[42] Lee E.S. (2014). Imaging diagnosis of pancreatic cancer: A state-of-the-art review. World J. Gastroenterol..

[43] Zamboni G.A., Kruskal J.B., Vollmer C.M., Baptista J., Callery M.P., Raptopoulos V.D. (2007). Pancreatic adenocarcinoma: Value of multidetector CT angiography in preoperative evaluation. Radiology.

[44] Califf R.M. (2018). Biomarker definitions and their applications. Exp. Biol. Med..

[45] Korde A., Patt M., Selivanova S.V., Scott A.M., Hesselmann R., Kiss O., Ramamoorthy N., Todde S., Rubow S.M., Gwaza L. (2024). Position Paper to Facilitate Patient Access to Radiopharmaceuticals: Considerations for a Suitable Pharmaceutical Regulatory Framework. EJNMMI Radiopharm. Chem..

[46] Jaymand M., Taghipour Y.D., Rezaei A., Derakhshankhah H., Abazari M.F., Samadian H., Hamblin M.R. (2021). Radiolabeled carbon-based nanostructures: New radiopharmaceuticals for cancer therapy?. Coord. Chem. Rev..

[47] Baskar R., Dai J., Wenlong N., Yeo R., Yeoh K.-W. (2014). Biological response of cancer cells to radiation treatment. Front. Mol. Biosci..

[48] Carvalho B.P.S.A., Frasson A.L., Santos M.M., de Barros N. (2011). Mammography findings following electron intraoperative radiotherapy or external radiotherapy for breast cancer treatment. Eur. J. Radiol..

[49] Boschi A., Uccelli L., Martini P. (2019). A Picture of Modern Tc-99m Radiopharmaceuticals: Production, Chemistry, and Applications in Molecular Imaging. Appl. Sci..

[50] Lepareur N., Ramée B., Mougin-Degraef M., Bourgeois M. (2023). Clinical advances and perspectives in targeted radionuclide therapy. Pharmaceutics.

[51] Alauddin M.M. (2012). Positron emission tomography (PET) imaging with ^18^F-based radiotracers. Am. J. Nucl. Med. Mol. Imaging.

[52] George S.C., Samuel E.J.J. (2023). Developments in ^177^Lu-based radiopharmaceutical therapy and dosimetry. Front. Chem..

[53] Pouget J.P., Santoro L., Raymond L., Chouin N., Bardiès M., Bascoul-Mollevi C., Huguet H., Azria D., Kotzki P.O., Pèlegrin M. (2008). Cell membrane is a more sensitive target than cytoplasm to dense ionization produced by Auger electrons. Radiat. Res..

[54] Fernandes C., Palma E., Silva F., Belchior A., Pinto C.I.G., Guerreiro J.F., Botelho H.M., Mendes F., Raposinho P., Paulo A. (2022). Searching for a Paradigm Shift in Auger-Electron Cancer Therapy with Tumor-Specific Radiopeptides Targeting the Mitochondria and/or the Cell Nucleus. Int. J. Mol. Sci..

[55] Pirovano G., Wilson T.C., Reiner T. (2021). Auger: The future of precision medicine. Nucl. Med. Biol..

[56] Salih S., Alkatheeri A., Alomaim W., Elliyanti A. (2022). Radiopharmaceutical Treatments for Cancer Therapy, Radionuclides Characteristics, Applications, and Challenges. Molecules.

[57] Merola E., Grana C.M. (2023). Peptide Receptor Radionuclide Therapy (PRRT): Innovations and Improvements. Cancers.

[58] Bodei L., Pepe G., Paganelli G. (2010). Peptide receptor radionuclide therapy (PRRT) of neuroendocrine tumors with somatostatin analogues. Eur. Rev. Med. Pharmacol. Sci..

[59] Pijeira M.S.O., Viltres H., Kozempel J., Sakmár M., Vlk M., İlem-Özdemir D., Ekinci M., Srinivasan S., Rajabzadeh A.R., Ricci-Junior E. (2022). Radiolabeled nanomaterials for biomedical applications: Radiopharmacy in the era of nanotechnology. EJNMMI Radiopharm. Chem..

[60] Milenic D.E., Brady E.D., Brechbiel M.W. (2004). Antibody-targeted radiation cancer therapy. Nat. Rev. Drug Discov..

[61] St. James S., Bednarz B., Benedict S., Buchsbaum J.C., Dewaraja Y., Frey E., Hobbs R., Grudzinski J., Roncali E., Sgouros G. (2021). Current Status of Radiopharmaceutical Therapy. Int. J. Radiat. Oncol. Biol. Phys..

[62] Borges de Souza P., McCabe C.J. (2021). Radioiodine treatment: An historical and future perspective. Endocr.-Relat. Cancer.

[63] Mayson S.E., Chan C.M., Haugen B.R. (2021). Tailoring the approach to radioactive iodine treatment in thyroid cancer. Endocr.-Relat. Cancer.

[64] Sgouros G., Bodei L., McDevitt M.R., Nedrow J.R. (2020). Radiopharmaceutical therapy in cancer: Clinical advances and challenges. Nat. Rev. Drug Discov..

[65] Sgouros G., Goldenberg D.M. (2014). Radiopharmaceutical therapy in the era of precision medicine. Eur. J. Cancer.

[66] Höllriegl V., Petoussi-Henss N., Hürkamp K., Ocampo Ramos J.C., Li W.B. (2021). Radiopharmacokinetic modelling and radiation dose assessment of ^223^Ra used for treatment of metastatic castration-resistant prostate cancer. EJNMMI Phys..

[67] Poeppel T.D., Handkiewicz-Junak D., Andreeff M., Becherer A., Bockisch A., Fricke E., Geworski L., Heinzel A., Krause B.J., Krause T. (2018). EANM guideline for radionuclide therapy with radium-223 of metastatic castration-resistant prostate cancer. Eur. J. Nucl. Med. Mol. Imaging.

[68] Poty S., Francesconi L.C., McDevitt M.R., Morris M.J., Lewis J.S. (2018). *α*-Emitters for Radiotherapy: From Basic Radiochemistry to Clinical Studies—Part 1. J. Nucl. Med..

[69] Baidoo K.E., Yong K., Brechbiel M.W. (2013). Molecular Pathways: Targeted *α*-Particle Radiation Therapy. Clin Cancer Res..

[70] Søyland C., Hassfjell S.P. (2000). Survival of human lung epithelial cells following in vitro *α*-particle irradiation with absolute determination of the number of alpha-particle traversals of individual cells. Int. J. Radiat. Biol..

[71] Zhou H., Hong M., Chai Y., Hei T.K. (2009). Consequences of Cytoplasmic Irradiation: Studies from Microbeam. J. Radiat. Res..

[72] Strosberg J., El-Haddad G., Wolin E., Hendifar A., Yao J., Chasen B., Mittra E., Kunz P.L., Kulke M.H., Jacene H. (2017). Phase 3 trial of 177Lu-dotatate for midgut neuroendocrine tumors. N. Engl. J. Med..

[73] Tickner B.J., Stasiuk G.J., Duckett S.B., Angelovski G. (2020). The use of yttrium in medical imaging and therapy: Historical background and future perspectives. Chem. Soc. Rev..

[74] Buchegger F., Perillo-Adamer F., Dupertuis Y.M., Bischof Delaloye A. (2006). Auger radiation targeted into DNA: A therapy perspective. Eur. J. Nucl. Med. Mol. Imaging.

[75] IRCCS Humanitas Research Hospital. https://www.humanitas.it/malattie/tumore-del-pancreas/.

[76] eMedicine. https://emedicine.medscape.com/article/2007067-overview?form=fpf.

[77] Conroy T., Pfeiffer P., Vilgrain V., Lamarca A., Seufferlein T., O’Reilly E.M., Hackert T., Golan T., Prager G., Haustermans K. (2023). Pancreatic cancer: ESMO Clinical Practice Guideline for diagnosis, treatment and follow-up. Ann. Oncol..

[78] Loharkar S., Basu S. (2023). Peptide receptor radionuclide therapy in neuroendocrine neoplasms and related tumors: From fundamentals to personalization and the newer experimental approaches. Expert Rev. Precis. Med. Drug Dev..

[79] Das S., Al-Toubah T., El-Haddad G., Strosberg J. (2019). ^177^Lu-DOTATATE for the treatment of gastroenteropancreatic neuroendocrine tumors. Expert Rev. Gastroenterol. Hepatol..

[80] Öberg K.E., Reubi J.-C., Kwekkeboom D.J., Krenning E.P. (2010). Role of Somatostatins in Gastroenteropancreatic Neuroendocrine Tumor Development and Therapy. Gastroenterology.

[81] Zaknun J.J., Bodei L., Mueller-Brand J., Baum R.P., Pavel M.E., Hörsch D., O’Dorisio M.S., O’Dorisio T.M., Howe J.R., Cremonesi M. (2013). The joint IAEA, EANM, and SNMMI practical guidance on peptide receptor radionuclide therapy (PRRNT) in neuroendocrine tumours. Eur. J. Nucl. Med. Mol. Imaging.

[82] Kim J.Y., Kim J., Kim Y., Yang D.H., Yoo C., Park I.J., Ryoo B.Y., Ryu J.S., Hong S.M. (2024). Somatostatin receptor 2 (SSTR2) expression is associated with better clinical outcome and prognosis in rectal neuroendocrine tumors. Sci. Rep..

[83] Basu S., Parghane R., Ranade R., Thapa P., Ramaswamy A., Ostwal V., Sirohi B., Panda D., Shrikhande S. (2019). Peptide receptor radionuclide therapy in the management of neuroendocrine tumors (Neoplasms)*: Fundamentals and salient clinical practice points for medical oncologists. Indian J. Med. Paediatr. Oncol..

[84] Yao J.C., Shah M.H., Ito T., Bohas C.L., Wolin E.M., Van Cutsem E., Hobday T.J., Okusaka T., Capdevila J., de Vries E.G.E. (2011). Everolimus for Advanced Pancreatic Neuroendocrine Tumors. N. Engl. J. Med..

[85] Yao J.C., Fazio N., Singh S., Buzzoni R., Carnaghi C., Wolin E., Tomasek J., Raderer M., Lahner H., Voi M. (2016). Everolimus for the Treatment of Advanced, Non-functional Neuroendocrine Tumours of the Lung or Gastrointestinal Tract (RADIANT-4): A Randomised, Placebo-Controlled, Phase 3 Study. Lancet.

[86] Raymond E., Dahan L., Raoul J.L., Bang Y.J., Borbath I., Lombard-Bohas C., Valle J., Metrakos P., Smith D., Vinik A. (2011). Sunitinib Malate for the Treatment of Pancreatic Neuroendocrine Tumors. N. Engl. J. Med..

[87] Pouget J.-P., Georgakilas A.G., Ravanat J.-L. (2018). Targeted and off-target (bystander and abscopal) effects of radiation therapy: Redox mechanisms and risk/benefit analysis. Antioxidants Redox Signal..

[88] Hull A., Li Y., Bartholomeusz D., Hsieh W., Allen B., Bezak E. (2020). Radioimmunotherapy of Pancreatic Ductal Adenocarcinoma: A Review of the Current Status of Literature. Cancers.

[89] Jiao R., Allen K.J.H., Malo M.E., Helal M., Jiang Z., Smart K., Buhl S.V., Rickles D., Bryan R.A., Dadachova E. (2019). Evaluation of novel highly specific antibodies to cancer testis antigen Centrin-1 for radioimmunoimaging and radioimmunotherapy of pancreatic cancer. Cancer Med..

[90] Aung W., Tsuji A.B., Sudo H., Sugyo A., Furukawa T., Ukai Y., Kurosawa Y., Saga T. (2016). Immunotargeting of Integrin *α*6*β*4 for Single-Photon Emission Computed Tomography and Near-Infrared Fluorescence Imaging in a Pancreatic Cancer Model. Mol. Imaging.

[91] Cruz-Monserrate Z., Qiu S., Evers B.M., O’Connor K.L. (2007). Upregulation and redistribution of integrin alpha6beta4 expression occurs at an early stage in pancreatic adenocarcinoma progression. Mod. Pathol..

[92] Kurosawa G., Akahori Y., Morita M., Sumitomo M., Sato N., Muramatsu C., Eguchi K., Matsuda K., Takasaki A., Tanaka M. (2008). Comprehensive screening for antigens overexpressed on carcinomas via isolation of human mAbs that may be therapeutic. Proc. Natl. Acad. Sci. USA.

[93] Hausner S.H., Bold R.J., Cheuy L.Y., Chew H.K., Daly M.E., Davis R.A., Foster C.C., Kim E.J., Sutcliffe J.L. (2019). Preclinical Development and First-in-Human Imaging of the Integrin *αvβ*6 with [^18^*F*]*αvβ*6-Binding Peptide in Metastatic Carcinoma. Clin. Cancer Res..

[94] Yoshii Y., Matsumoto H., Yoshimoto M., Oe Y., Zhang M.R., Nagatsu K., Sugyo A., Tsuji A.B., Higashi T. (2019). ^64^Cu-Intraperitoneal Radioimmunotherapy: A Novel Approach for Adjuvant Treatment in a Clinically Relevant Preclinical Model of Pancreatic Cancer. J. Nucl. Med..

[95] Manzur A., Oluwasanmi A., Moss D., Curtis A., Hoskins C. (2017). Nanotechnologies in Pancreatic Cancer Therapy. Pharmaceutics.

[96] Myung J.H., Tam K.A., Park S., Cha A., Hong S. (2016). Recent advances in nanotechnology-based detection and separation of circulating tumor cells. Wiley Interdiscip. Rev. Nanomed. Nanobiotechnol..

[97] Greish K. (2010). Enhanced permeability and retention (EPR) effect for anticancer nanomedicine drug targeting. Methods Mol. Biol..

[98] Byrne J.D., Betancourt T., Brannon-Peppas L. (2008). Active targeting schemes for nanoparticle systems in cancer therapeutics. Adv. Drug Deliv. Rev..

[99] Goodman T.T., Chen J., Matveev K., Pun S.H. (2008). Spatio-temporal modeling of nanoparticle delivery to multicellular tumor spheroids. Biotechnol. Bioeng..

[100] Chauhan V.P., Popovi’c Z., Chen O., Cui J., Fukumura D., Bawendi M.G., Jain R.K. (2011). Fluorescent nanorods and nanospheres for real-time in vivo probing of nanoparticle shape-dependent tumor penetration. Angew. Chem..

[101] Yuan F., Leunig M., Huang S.K., Berk D.A., Papahadjopoulos D., Jain R.K. (1994). Microvascular permeability and interstitial penetration of sterically stabilized (stealth) liposomes in a human tumor xenograft. Cancer Res..

[102] Trujillo-Nolasco M., Cruz-Nova P., Ferro-Flores G., Gibbens-Bandala B., Morales-Avila E., Aranda-Lara L., Vargas M., Ocampo-García B. (2021). Development of ^177^Lu-DN(C19)-CXCR4 Ligand Nanosystem for Combinatorial Therapy in Pancreatic Cancer. J. Biomed. Nanotechnol..

[103] Singh S., Srivastava S.K., Bhardwaj A., Owen L.B., Singh A.P. (2010). CXCL12–CXCR4 signalling axis confers gemcitabine resistance to pancreatic cancer cells: A novel target for therapy. Br. J. Cancer.

[104] Jalilian A.R., Ocampo-García B., Pasanphan W., Sakr T.M., Melendez-Alafort L., Grasselli M., Lugao A.B., Yousefnia H., Dispenza C., Mohd Janib S. (2022). IAEA Contribution to Nanosized Targeted Radiopharmaceuticals for Drug Delivery. Pharmaceutics.

[105] Imran M., Arshad M.S., Butt M.S., Kwon J.H., Arshad M.U., Sultan M.T. (2017). Mangiferin: A natural miracle bioactive compound against lifestyle related disorders. Lipids Health Dis..

[106] Al-Yasiri A.Y., Khoobchandani M., Cutler C.S., Watkinson L., Carmack T., Smith C.J., Kuchuk M., Loyalka S.K., Lugao A.B., Katti K.V. (2017). Mangiferin functionalized radioactive gold nanoparticles (MGF-(198)AuNPs) in prostate tumor therapy: Green nanotechnology for production, in vivo tumor retention and evaluation of therapeutic efficacy. Dalton Trans..

[107] Khoobchandani M., Katti K.K., Karikachery A.R., Thipe V.C., Srisrimal D., Mohandoss D.K.D., Darshakumar R.D., Joshi C.M., Katti K.V. (2020). New approaches in breast cancer therapy through green nanotechnology and nano-ayurvedic medicine–pre-clinical and pilot human clinical investigations. Int. J. Nanomed..

[108] Khoobchandani M., Khan A., Katti K.K., Thipe V.C., Al-Yasiri A.Y., MohanDoss D.K., Nicholl M.B., Lugão A.B., Hans C.P., Katti K.V. (2021). Green nanotechnology of MGF-AuNPs for immunomodulatory intervention in prostate cancer therapy. Sci. Rep..

[109] Bannik K., Madas B., Jarzombek M., Sutter A., Siemeister G., Mumberg D., Zitzmann-Kolbe S. (2019). Radiobiological effects of the alpha emitter Ra-223 on tumor cells. Sci. Rep..

[110] Burkett B.J., Bartlett D.J., McGarrah P.W., Lewis A.R., Johnson D.R., Berberoğlu K., Pandey M.K., Packard A.T., Halfdanarson T.R., Hruska C.B. (2023). A Review of Theranostics: Perspectives on Emerging Approaches and Clinical Advancements. Radiol. Imaging Cancer.

[111] Watabe T., Kabayama K., Naka S., Yamamoto R., Kaneda K., Serada S., Ooe K., Toyoshima A., Wang Y., Haba H. (2023). Immuno-PET and Targeted *α*-Therapy Using Anti–Glypican-1 Antibody Labeled with ^89^Zr or ^211^At: A Theranostic Approach for Pancreatic Ductal Adenocarcinoma. J. Nucl. Med..

[112] Liu L., Kshirsagar P.G., Gautam S.K., Gulati M., Wafa E.I., Christiansen J.C., White B.M., Mallapragada S.K., Wannemuehler M.J., Kumar S. (2022). Nanocarriers for pancreatic cancer imaging, treatments, and immunotherapies. Theranostics.

[113] Wang L., An Y., Yuan C., Zhang H., Liang C., Ding F., Gao Q., Zhang D. (2015). GEM-loaded magnetic albumin nanospheres modified with cetuximab for simultaneous targeting, magnetic resonance imaging, and double-targeted thermochemotherapy of pancreatic cancer cells. Int. J. Nanomed..

[114] Liu F., Zhu H., Yu J., Han X., Xie Q., Liu T., Xia C., Li N., Yang Z. (2017). ^68^Ga/^177^Lu-labeled DOTA-TATE shows similar imaging and biodistribution in a neuroendocrine tumor model. Tumour Biol..

[115] Davis R., Foster C., Ganguly T., Hausner S., Kim E., Roncali E., Sutcliffe J. (2023). First-in-human study of the theranostic pair [^68^Ga]GaDOTA-5G and [^177^Lu]LuDOTA-ABM-5G in pancreatic adenocarcinoma. J. Nucl. Med..

[116] Han H.S., Choi K.Y. (2021). Advances in Nanomaterial-Mediated Photothermal Cancer Therapies: Toward Clinical Applications. Biomedicines.

[117] Shi X., Li Q., Zhang C., Pei H., Wang G., Zhou H., Fan L., Yang K., Jiang B., Wang F. (2021). Semiconducting polymer nano-radiopharmaceutical for combined radio-photothermal therapy of pancreatic tumor. J. Nanobiotechnol..

[118] McHale A.P., Callan J.F., Nomikou N., Fowley C., Callan B., Escoffre J.M., Bouakaz A. (2016). Sonodynamic Therapy: Concept, Mechanism and Application to Cancer Treatment. Therapeutic Ultrasound.

[119] An J., He X., Ma H., Li Y., Li Y., Zhang X., Shuai Q., Wang Y., Liu W., Li W. (2023). Radionuclide labeled nanocarrier for imaging guided combined radionuclide, sonodynamic, and photothermal therapy of pancreatic tumours. J. Colloid Interface Sci..

[120] Duan H., Li L., He S. (2023). Advances and Prospects in the Treatment of Pancreatic Cancer. Int. J. Nanomed..

[121] Hu Y.Y., Chi C.W., Wang S.H., Wang L.X., Liang P., Liu F.Y., Shang W.T., Wang W.W., Zhang F.R., Li S.S. (2017). A Comparative Study of Clinical Intervention and Interventional Photothermal Therapy for Pancreatic Cancer. Adv. Mater..

[122] Manafi-Farid R., Ataeinia B., Ranjbar S., Jamshidi Araghi Z., Moradi M.M., Pirich C., Beheshti M. (2022). ImmunoPET: Antibody-Based PET Imaging in Solid Tumors. Front. Med..

[123] González-Gómez R., Pazo-Cid R.A., Sarría L., Morcillo M., Schuhmacher A.J. (2021). Diagnosis of Pancreatic Ductal Adenocarcinoma by Immuno-Positron Emission Tomography. J. Clin. Med..

[124] Sawada R., Sun S.-M., Wu X., Hong F., Ragupathi G., Livingston P.O., Scholz W.W. (2011). Human Monoclonal Antibodies to Sialyl-Lewisa (CA19.9) with Potent CDC, ADCC, and Antitumor Activity. Clin. Cancer Res..

[125] Viola-Villegas N.T., Rice S.L., Carlin S., Wu X., Evans M.J., Sevak K.K., Drobjnak M., Ragupathi G., Sawada R., Scholz W.W. (2013). Applying PET to Broaden the Diagnostic Utility of the Clinically Validated CA19.9 Serum Biomarker for Oncology. J. Nucl. Med..

[126] Lohrmann C., O’Reilly E.M., O’Donoghue J.A., Pandit-Taskar N., Carrasquillo J.A., Lyashchenko S.K., Ruan S., Teng R., Scholz W., Maffuid P.W. (2019). Retooling a Blood-Based Biomarker: Phase I Assessment of the High-Affinity CA19-9 Antibody HuMab-5B1 for Immuno-PET Imaging of Pancreatic Cancer. Clin. Cancer Res..

[127] Roehrich M., Preussig M., Lang M., Schroeter C., Gutjahr E., Schreckenberger M., Haberkorn U. (2024). Diagnostic potential of static and dynamic ^68^Ga-FAPI PET/CT for the differentiation of mass forming pancreatitis and pancreatic ductal adenocarcinomas. J. Nucl. Med..

[128] Nikolova E., Tonev D., Zhelev N., Neychev V. (2021). Prospects for Radiopharmaceuticals as Effective and Safe Therapeutics in Oncology and Challenges of Tumor Resistance to Radiotherapy. Dose-Response.

[129] Cheng J.C., Bai A., Beckham T.H., Marrison S.T., Yount C.L., Young K., Lu P., Bartlett A.M., Wu B.X., Keane B.J. (2013). Radiation-induced acid ceramidase confers prostate cancer resistance and tumor relapse. J. Clin. Investig..

[130] Ge X., Su L., Chen Z., Zhu K., Zhang X., Wu Y., Song J. (2023). A Radio-Pharmaceutical Fluorescent Probe for Synergistic Cancer Radiotherapy and Ratiometric Imaging of Tumor Reactive Oxygen Species. Angew. Chem. Int. Ed..

[131] Krishnaraj J., Yamamoto T., Ohki R. (2023). p53-Dependent Cytoprotective Mechanisms behind Resistance to Chemo-Radiotherapeutic Agents Used in Cancer Treatment. Cancers.

[132] Wu Y., Song Y., Wang R., Wang T. (2023). Molecular mechanisms of tumor resistance to radiotherapy. Mol. Cancer.

[133] Ramaekers M., Viviers C.G.A., Janssen B.V., Hellström T.A.E., Ewals L., van der Wulp K., Nederend J., Jacobs I., Pluyter J.R., Mavroeidis D. (2023). Computer-Aided Detection for Pancreatic Cancer Diagnosis: Radiological Challenges and Future Directions. J. Clin. Med..

